# Single-Grain Quartz OSL Characteristics: Testing for Correlations within and between Sites in Asia, Europe and Africa

**DOI:** 10.3390/mps3010002

**Published:** 2019-12-26

**Authors:** Yue Hu, Bo Li, Zenobia Jacobs

**Affiliations:** 1 Department of Archaeology, School of History and Culture, Sichuan University, Chengdu 610207, China; 2 Centre for Archaeological Science, School of Earth and Environmental Science, University of Wollongong, Wollongong, NSW 2522, Australia; bli@uow.edu.au (B.L.); zenobia@uow.edu.au (Z.J.); 3 Australian Research Council (ARC) Centre of Excellence for Australian Biodiversity and Heritage, University of Wollongong, Wollongong, NSW 2522, Australia

**Keywords:** OSL, quartz, standardized growth curves, decay curve, saturation dose

## Abstract

We studied the characteristics of the optically stimulated luminescence (OSL) signal of single-grain quartz from three sites in China, Italy, and Libya, including the brightness, decay curve and dose response curve (DRC) shapes, recuperation, and reproducibility. We demonstrate the large variation in OSL behaviors for individual quartz grains of different samples from different regions, and show that recuperation, sensitivity change, and reproducibility are independent of the brightness and decay curve shape of the OSL signals. The single-grain DRCs can be divided into at least eight groups with different characteristic saturation doses (D_0_), and a standardized growth curve (SGC) can be established for each of the DRC groups. There is no distinctive difference in the shape of OSL decay curves among different DRC groups, but samples from different regions have a difference in the OSL sensitivities and decay shapes for different groups. Many of the quartz grains have low D_0_ values (30–50 Gy), and more than 99% of the grains have D_0_ values of <200 Gy. Our results raise caution against the dating of samples with equivalent dose values higher than 100 Gy, if there are many low-D_0_ and ‘saturated’ grains.

## 1. Introduction

Single-grain optically stimulated luminescence (OSL) dating of quartz has been widely used for dating sediments, because of its inherent advantages over the single-aliquot method in identifying poorly behaved grains and dealing with insufficiently bleached samples and those affected by post-depositional mixing (e.g., [1,2,3,4,5]). It has previously been reported that different quartz grains may have substantially different OSL behaviors even for grains from the same sample or site (e.g., [6,7,8]). Understanding the intrinsic variability of OSL behaviors at the single-grain level is important for dating, since D_e_ estimation can be dependent on these behaviors, e.g., the shape of the dose response curve (DRC) (or characteristic saturation dose, D_0_) [9,10,11,12,13,14,15], OSL sensitivity (or brightness) [16], and shape of OSL decay curves [17,18,19].

Previous studies have focused on the relationship between D_e_ estimates and the variability of a particular OSL characteristic for single grains, e.g., luminescence sensitivity [1,7,15,20], shape of OSL decay curve [1,6,7,15,19,20], and measurement uncertainties (including counting errors and instrument irreproducibility errors) [21]. However, none of these studies have systematically investigated correlations between multiple luminescence behaviors. In this paper, we studied quartz grains extracted from three sites in China, Italy, and Libya. We compared the OSL characteristics of different grains from the same and different samples from different sites. We investigated the relationship and correlation between the OSL characteristics, and discuss their implications for optical dating.

## 2. Sample Description, Preparation, and Measurements

Seven sediment samples were studied, including three from the Tianhuadong Cave (THD) [22], Yunnan Province, southwest China; one from the Visogliano (VISO) in Italy [23]; and three from the Haua Fteah Cave (HF) in Libya [14,24]. The samples from THD and HF have been described in detail in Hu et al. [25] and Jacobs et al. [14], respectively. These sites were chosen because their quartz grains generally have bright OSL signals, which allows us to study and compare their luminescence behavior in detail. Quartz grains of 150 to 180 or 180 to 212 µm in diameter were extracted using standard preparation procedures [26,27].

All OSL measurements were made on automated Risø TL-DA-20 luminescence readers equipped with a green laser (532 nm) [28]. Laboratory irradiations were carried out within the luminescence readers using calibrated ^90^Sr/^90^Y beta sources. For single-grain OSL measurements, standard Risø single grain discs were used (each disc contains 100 holes, each 300 μm in diameter and 300 μm deep) [29]. For the samples of 150 to 180 µm in diameter, extra care was taken to ensure that each hole contained only one grain (e.g., by picking up extra grains with a needle of static electricity). The discs were visually checked under a microscope to ensure that each hole contained only one grain. The spatial variation in the dose rate for individual grain positions was calibrated using gamma-irradiated calibration quartz standards. For the readers used in this study, the maximum difference of single-grain dose rates ranged from ~30% to ~60% (with relative standard deviation from ~5% to ~15%). The ultraviolet OSL emissions were detected by an Electron Tubes Ltd. 9235QA photomultiplier tube fitted with a 7.5-mm Hoya U-340 filter.

A single-aliquot regenerative-dose (SAR) procedure [30,31] was applied to measure the OSL signals and establish DRCs for individual grains. The SAR procedure (Table S1) involves measuring the OSL signals from the natural (burial) dose and from a series of regenerative doses, each of which was preheated at a specific temperature (240 °C) for 10 s prior to optical stimulation by the green laser beam for 2 s at 125 °C. A fixed test dose (D_t_ = ~10 Gy) was given after each natural and regenerative dose, with the induced test dose OSL signals used to monitor any sensitivity change that may have occurred during the SAR sequence. A cutheat to a temperature (180 °C) was applied to the test dose. From seven to nine regenerative doses (up to ~1200 Gy) were measured for each sample, including a duplicate regenerative dose to check on the validity of sensitivity correction and a ‘zero dose’ to monitor the extent of any ‘recuperation’ or ‘thermal transfer’ induced by the preheating. We also applied the OSL IR depletion-ratio test [32] at the end of the SAR sequence, using an infrared bleach of 40 s at 50 °C, to check for feldspar contamination.

## 3. Comparing OSL Characteristics

### 3.1. OSL Decay Curves and Signal Intensities

**Figure 1:**
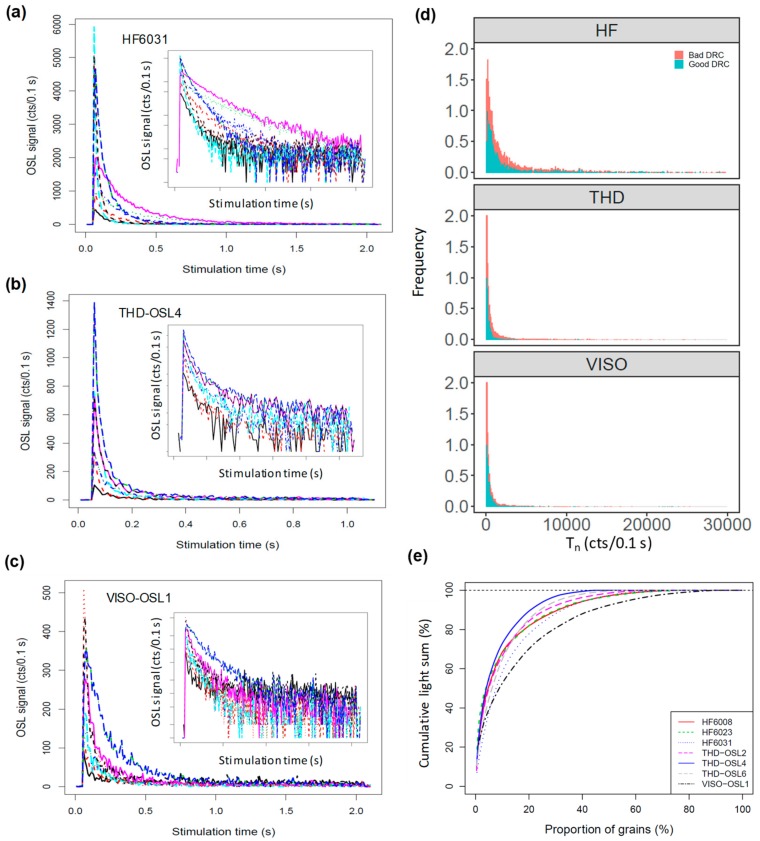
Ten representative OSL decay curves from three samples (**a**) HF6031, (**b**) THD-OSL4, and (**c**) VISO-OSL1. The inset panels show the same curves but with the *y*-axis on a logarithmic scale. (**d**) The density distribution of the test dose signal intensity (T_n_) of individual grains from each site. Different colors represent the grains associated with poor or good DRCs, respectively (see Section 3.2). (**e**) Cumulative light sum plotted against the proportion of grains for each sample.

We first investigated the OSL decay signals from individual grains for different samples. Figure 1a–c shows the test-dose OSL decay curves of 10 grains for one sample (HF6031, THD-OSL4, VISO-OSL1) from each of the three sites. It is shown that most of the OSL signals decay to negligible levels after 0.5 s of green-laser stimulation. We calculated the net OSL intensity based on the integral from the first 0.1 s of OSL decay with subtraction of the background estimated from the last 0.3 s. The OSL intensity varies significantly from grain to grain. The net OSL signal intensities of the first test dose (T_n_) range from a few tens to several tens of thousands of counts per 0.1 s of stimulation time (Figure 1d). The samples from HF have much brighter grains and a wider range of T_n_ intensities, followed by those from THD. In contrast, the samples from VISO are comparatively dimmer and have a much narrower range of T_n_ intensities. We calculated the cumulative percentage of luminescence intensities for all the samples, and it is observed that for most of the samples, 15% to 20% of the grains contribute ~80% of the total luminescence (Figure 1e).

**Figure 2:**
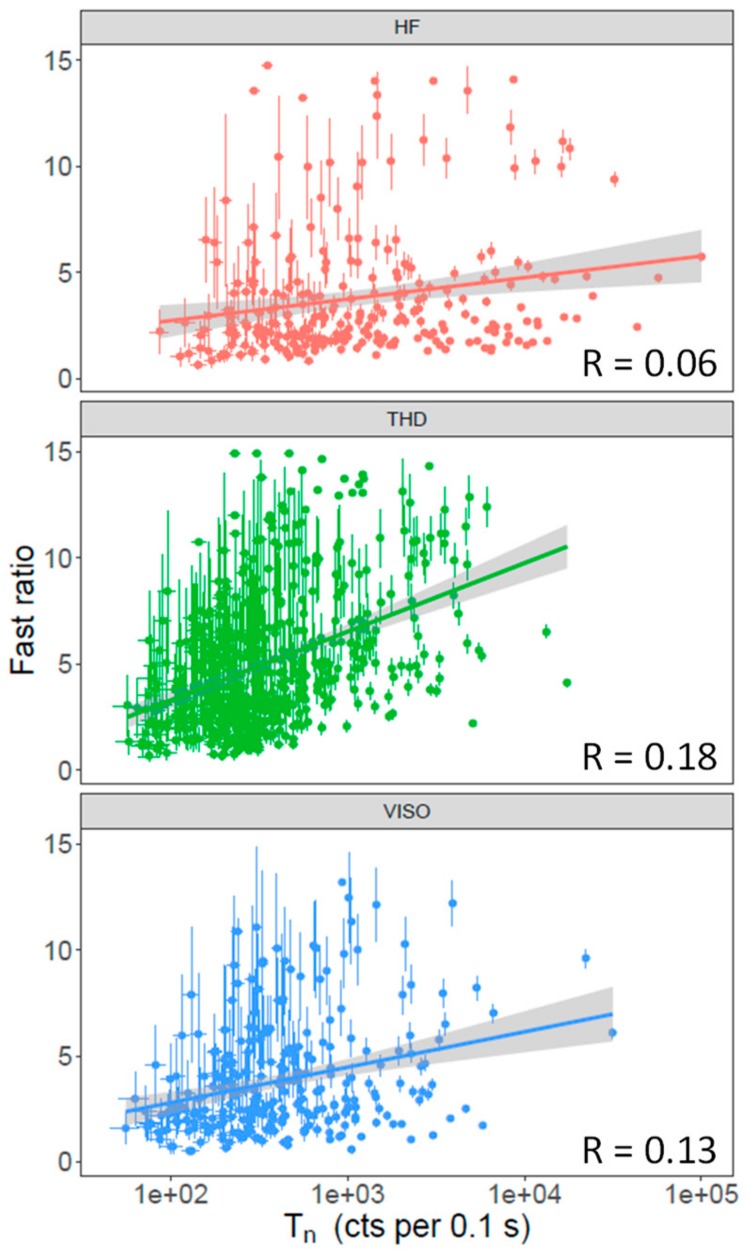
Relationship between the test dose signal intensity (T_n_) and fast ratio of individual grains from each site.

To investigate if there was any correlation between the OSL signal intensity and decay curve shape, we compared the fast ratios (FR) [33,34]—A quantitative measure of the dominance of the fast component in the initial OSL signal—of the T_n_ signal against its intensities. The fast ratios were calculated using the following equation:

(1) $$\mathrm{Fast}\;\mathrm{Ratio}=\frac{F-S}{M-S}$$

where F is the initial intensity of the signal from the first 0.02 s of the decay curve, M is the signal intensity from 0.07 to 0.08 s, and S represents the contribution of slow components and background estimated from the signals from 0.5 to 0.6 s. Figure 2 shows the relationship between T_n_ and FR for samples from each of the three sites. It appears that brighter grains tend to have larger FR ratios, which is indicated by the positive correlation coefficients (R values) and is especially prominent for samples from VISO (R = 0.13) and THD (R = 0.18), although many dimmer grains also have larger FR ratios and uncertainties. We also tested using a later integral (0.9–1.0 s) for S, and we found no detectable change in the pattern of the relationship. This result suggests that selecting brighter grains may preferentially select grains dominated by the fast component.

### 3.2. Dose Response Curves

Apart from the variation in the OSL decay curve shapes and intensities, we also investigated the variability of DRCs among grains and samples. Grains were rejected if they had one or more of the following properties: (1) Test-dose signal following the natural dose (T_n_) was too dim, i.e., the initial intensity was below the instrument detection limit (3σ below background intensity) and/or the relative standard error on the test dose measurement was more than 20%; (2) recuperation or thermal transfer was too high, i.e., the ratio between the sensitivity-corrected OSL signals for the zero dose and the largest regenerative dose was greater than 5%; and (3) the DRC data were too scattered to be fitted with suitable functions (e.g., a single saturating exponential function or a general-order kinetic (GOK) function [35]). We used a figure-of-merit (FOM) value of 10% and a reduced-chi-square (RCS) value of 5, as recommended by Peng and Li [36], as the upper limits for selecting satisfactory DRCs. The implementation of the rejection process was achieved using the functions provided in the R-package ‘numOSL’ [36]. The numbers of grains measured and rejected for each sample are summarized in Table 1. About 25%, 40%, and 65% of the grains from each of the three sites were rejected due to signals being too weak. Less than 5% of the grains in all samples had recuperation values greater than 5%. It is interesting to note that the proportions of grains with poor DRCs differed significantly from site to site, e.g., ~40% for HF, ~11% to 29% for THD, and 22% for VISO. Since the samples from HF are generally much brighter than those from the other sites, it is likely that the brighter grains may be preferentially rejected by the criterion of ‘poor DRC’. In order to test this, we compared the signal intensity distributions of the T_n_ signal from the grains being associated with ‘good’ and ‘bad’ DRCs, respectively (Figure 1d). It shows that there was no distinctive difference between the T_n_ intensity distributions of the two groups, indicating that the different proportions of grains with poor DRCs reflects the variability of the grain’s behavior among different sites.

**Figure 3:**
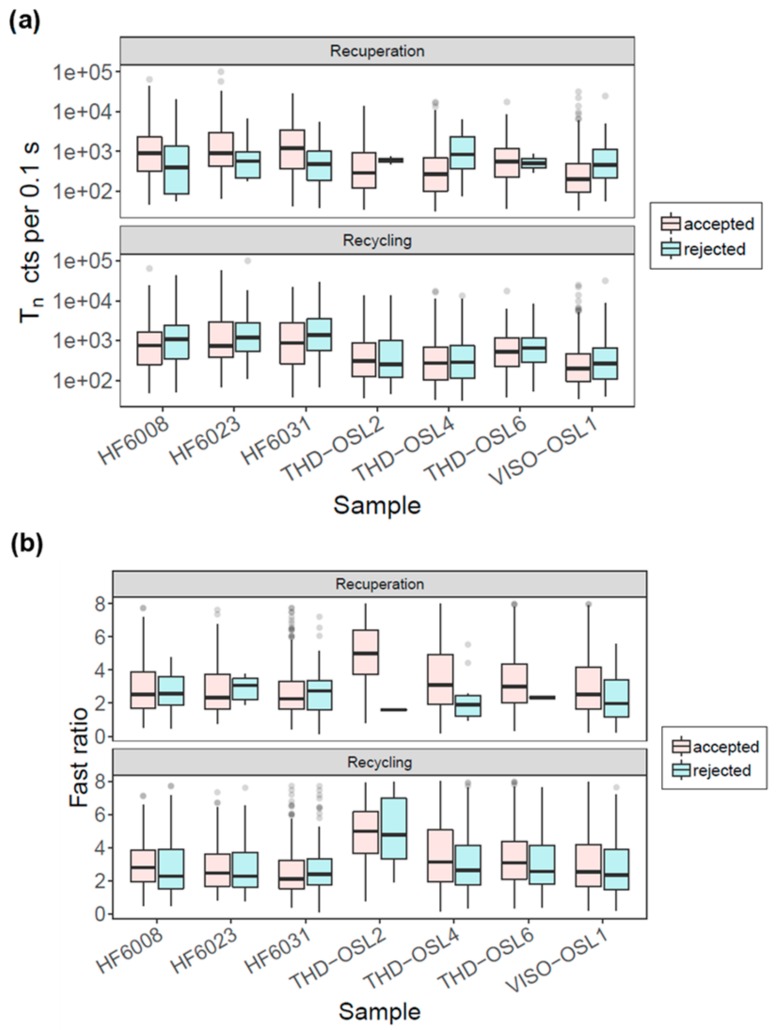
Boxplots showing the (**a**) sensitivity (T_n_) and (**b**) fast ratio of grains with different recuperation percentages and recycling ratios. Grains that have recuperation values <5% or recycling ratios consistent with unity (at 2σ) are shown as ‘accepted’ (light blue bars), and those with recuperation values >5% or recycling ratios inconsistent with unity are shown as ‘rejected’ (pink bars). The center lines in each of the boxes show the data median. Boxes show the first and third quartiles (the 25th and 75th percentiles), and the whiskers extend from the upper and lower hinge to the largest and smallest values no further than 1.5 times the interquartile range from the hinge. Data beyond the end of the whiskers are outliers and are plotted individually.

**Figure 4:**
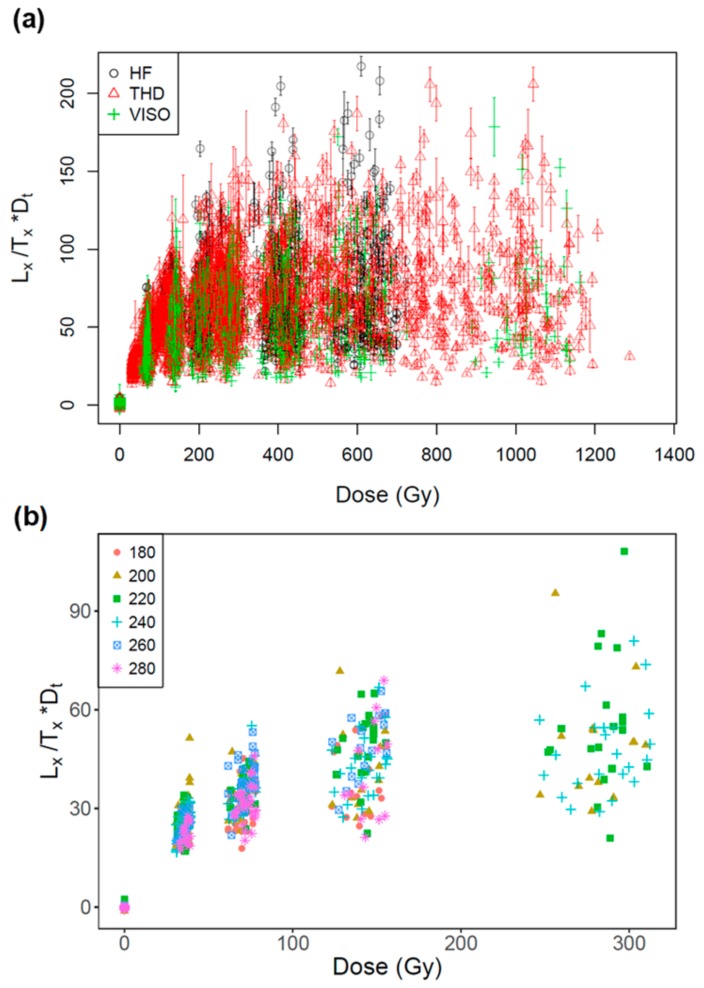
(**a**) Test-dose normalized sensitivity-corrected signal (L_x_/T_x_*D_t_) plotted as a function of regenerative doses for a total of 1146 grains from the study sites. (**b**) Comparison of DRCs obtained for the sample THD-OSL4 using different preheating temperatures (shown in different symbols).

To investigate whether the OSL signals from the rejected and accepted grains had distinctly different features, we compared the sensitivity (T_n_) and OSL decay curve shapes (using the FR as proxy) of grains to their recuperation value and recycling ratio. For recuperation, we compared all grains with recuperation values <5% (accepted) and >5% (rejected). For the recycling ratio, we compared the grains that had ratios consistent with unity at 2σ (accepted) and those that were inconsistent with unity (rejected). Figure 3a,b shows the T_n_ and FR values for all accepted and rejected grains based on either the recuperation or recycling ratio from different samples. No distinctive differences were observed. Figure 4 shows all test-dose normalized sensitivity-corrected signals (L_x_/T_x_*D_t_) from a total of 1146 grains that passed the rejection criteria. It shows large between-grain variability in DRCs from the same site, and a similar range of DRCs between grains from different sites. We also measured the DRCs for sample THD-OSL4 using different preheating temperatures ranging from 180 to 280 °C (Figure 4b), and no distinctive difference was observed in the variability in DRCs for different preheating temperatures. The results suggest that the variation in the DRCs is an intrinsic physical behavior rather than an artefact caused by the preheating conditions.

### 3.3. Sensitivity Change

**Figure 5:**
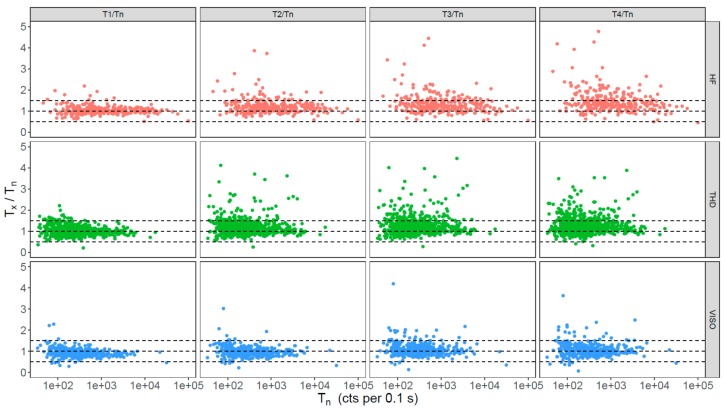
Ratios between test-dose signals (T_x_) of the second to fifth SAR cycles (T_1_–T_4_) and the first cycle (T_n_) plotted against inherent sensitivity (T_n_) for grains from different sites (shown in different rows). The dashed horizontal lines represent values at 0.5, 1.0, and 1.5, respectively.

Sensitivity change in quartz OSL are commonly observed during SAR measurements [37,38] and may be caused by irradiation, preheating, and/or OSL stimulation [39]. A reliable D_e_ determination relies on a successful correction of the sensitivity change, which is usually monitored through the use of a recycling ratio as part of the SAR procedure. Grains with poor recycling ratios are usually rejected for D_e_ analysis [1]. In this section, we investigated the extent of the sensitivity change for different grains during the SAR measurement procedure and its relationship to different OSL characteristics, such as the brightness, decay curve shape, recuperation, and reproducibility (e.g., recycling ratio). We used the ratio between T_x_ and T_n_ as an indicator of the extent of the sensitivity change through SAR cycles. Figure 5 plots the T_x_/T_n_ values obtained for all grains from each site. The test dose signals measured during the first five SAR cycles (including those corresponding to the natural dose, T_n_, and four regenerative doses, T_1_–T_4_) were normalized to T_n_. Most of the grains show some sensitivity change from cycle to cycle, and were sensitized or de-sensitized by <50% during the first two measurement cycles (T_1_/T_n_). Most of them, however, gradually sensitized during subsequent measurement cycles, indicated by the systematic increase in T_x_/T_n_ values. There are a small proportion of grains that produced a >100% change in sensitivity during two successive measurement cycles; this is most prominent for samples from HF and THD. Furthermore, the extent of sensitivity change is not significantly correlated to the sensitivity of the grains (Figure 5) or rate of decay of the OSL signals (Figure S1).

## 4. Grouping of Grains According to the Shape of Their DRCs

Li et al. [13] found that small-aliquot DRCs for samples from HF could be divided into three groups, which saturate at small, intermediate, and high doses, respectively. They proposed a method of grouping individual DRCs, by analyzing the ratio of L_x_/T_x_ values between two regenerative dose points, and used the least-square normalization (LS-normalization) procedure to establish a standardized growth curve (SGC) for each of the groups. To test the variability of DRCs of samples from different regions, we grouped and applied the LS-normalization procedure to all grains measured for all the samples investigated in this study.

One way of comparing the shapes of DRCs is to fit the individual curve data from different grains with a single saturating exponential function, f(x) = A [1 − exp(−x/D_0_)], where x is the dose, D_0_ is the characteristic saturation dose, and A is a constant, and then use the D_0_ estimate for comparison. However, there are several drawbacks to this method. Firstly, D_0_ estimates are usually imprecise when only a few regenerative data (e.g., 5–7) and a relatively narrow dose range (e.g., <2D_0_) are measured. Li et al. [21] demonstrated that the D_0_ estimates are influenced significantly by measurement uncertainties and also the measurement strategy (such as the number and range of regenerative doses applied). Another issue with D_0_ is that it only works when all the DRCs follow a single saturating exponential function, and it becomes problematic when there are many grains follow different growth patterns, such as double exponential or exponential plus linear. For this reason, we followed the method of Li et al. [13] by calculating the L_x_/T_x_ ratios to quantify the saturation characteristic (e.g., shape of DRC) of different grains. We chose the L_x_/T_x_ values of two regenerative doses, ~300 and ~70 Gy, respectively (see Li et al. [13] for a full discussion about how to choose the two regenerative doses). Since different grains have different regenerative doses due to the spatial variation in the dose rates of the beta sources, it is impossible to find exactly the same regenerative doses for all grains. To deal with this, we first fitted the measured L_x_/T_x_ data for individual grains using a GOK function, and then estimated the L_x_/T_x_ values at 300 and 70 Gy based on the best-fit DRCs for individual grains. In order to estimate the uncertainties of individual L_x_/T_x_ ratios, we applied a Monte Carlo method. This involved generating random L_x_/T_x_ values based on the experimental L_x_/T_x_ values and their uncertainties according to Gaussian distributions (by taking the uncertainty of each L_x_/T_x_ value as the standard deviation of the distribution). After that, the generated data were fitted using a GOK function, and an L_x_/T_x_ ratio was calculated for each simulation. This process was repeated 500 times, so that a total of 500 L_x_/T_x_ ratios were obtained. The final L_x_/T_x_ ratio estimate and its uncertainty were then derived directly from the mean and the standard deviation of the sampling distribution of the 500 ratios.

**Figure 6:**
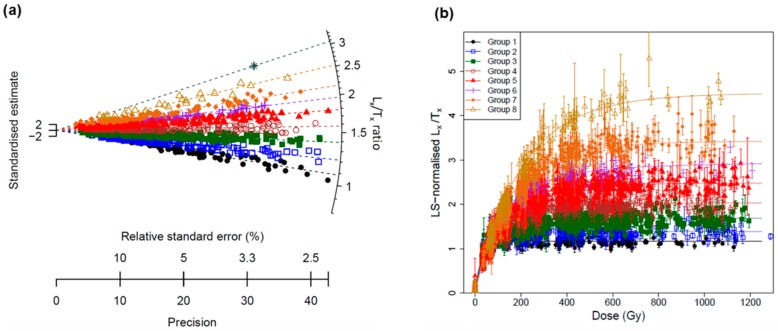
(**a**) Radial plot showing the distribution of the ratios of L_x_/T_x_ values between two regenerative doses of 300 and 70 Gy for all accepted grains. The different colors and symbols represent different groups of grains identified using the FMM. (**b**) The LS-normalized L_x_/T_x_ values plotted against regenerative doses for different groups. The data set for each group was fitted using a GOK function (full lines) and then normalized to unity at 50 Gy.

The ratios for all the grains are shown in Figure 6a. A large range of ratios from ~1 to ~3 was observed, indicating that the grains have a wide range of saturation doses. For example, the grains with L_x_/T_x_ ratios close to 1 correspond to early saturated grains (i.e., there was a negligible increase in the OSL signal beyond 70 Gy). In contrast, grains with higher L_x_/T_x_ ratios have a larger saturation dose level.

To find grains that share a similar saturation level, we applied the finite mixture model (FMM) [40,41,42] to analyze the ratios for all accepted grains. We ran the FMM using the build-in function from the R package ‘Luminescence’ [43] by setting the expected overdispersion (so-called sigma-b value) as zero (assuming that the sources of error associated with the signal intensity have been adequately taken into account) and increasing the number of groups from 3 to 10. The optimal number of groups was then estimated as the one associated with the lowest Bayesian information criterion. We found that nine groups is an optimum number needed to account for the observed spread in ratios for our samples (Figure 6a). As the ninth group (with the largest ratio of ~3) only contained one grain, we treated this grain as an outlier and ignored this group in all subsequent analyses.

We noted that the HF samples contained grains from all the eight groups identified in our study, which is different from the three groups identified by Li et al. [13] using the samples from the same sites. This is, however, expected as (1) this study is a true single-grain result while Li et al. [13] used a small aliquot (e.g., single-grain discs with smaller grains (90–125 um), where each hole contains about ~ eight grains); (2) the present study analyzed a much larger number of grains, which would reveal more detailed information; and (3) Li et al. [13] assumed an extra overdispersion value of 3.5% when applying FMM, but we assumed zero overdispersion in this study.

Since the L_x_/T_x_ ratios from two regenerative doses do not necessarily tell us the shape of DRC, it is still possible that two grains with the same L_x_/T_x_ ratios have different shaped DRC (e.g., one grain could follow a single-saturation exponential function, and the other could follow a single-saturation exponential plus linear function). Whether different grains share the same shape of DRCs can only be proved by establishing SGCs and investigating the goodness-of-fit of SGC to the data. In order to test whether the eight groups identified in Figure 6 actually did represent their difference in DRCs, the LS-normalization procedure was used to analyze the DRCs from each group. The GOK function was used to construct the DRCs for data from the same groups. Figure 6b shows the LS-normalized regenerative-dose data and their corresponding SGC curves for the eight groups. It can be seen that different groups have considerably different saturation dose levels. When the data for each group were fitted using a single saturating exponential function, D_0_ values of 32 ± 2, 39 ± 2, 57 ± 1, 76 ± 2, 97 ± 2, 120 ± 5, 144 ± 6, and 197 ± 12 Gy were calculated for groups 1 to 8. To test the validity of the groupings and establishment of the SGCs, the ratios between the measured L_x_/T_x_ values and the expected values based on the best-fitted SGCs were calculated and are shown in Figure S2. The results show that most of the measured-to-expected signal ratios (~90% or more) are statistically consistent with unity at 2σ for all the groups, indicating that most of the grains from the same groups classified based on the L_x_/T_x_ ratios follow the same DRC shape.

**Figure 7:**
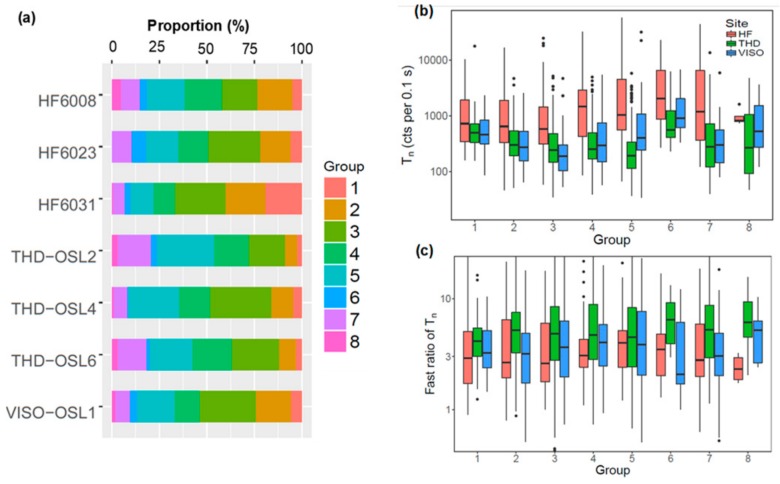
(**a**) Proportional distribution of accepted grains that make up the different DRC groups for each sample. (**b**) Boxplots showing the distribution of T_n_ for all grains from different DRC groups and different sites (shown as different colors). (**c**) Boxplots showing the fast ratio of T_n_ for all grains from different DRC groups and different sites (shown as different colors). Centre lines in each of the boxes show the data median. Boxes show the first and third quartiles (the 25th and 75th percentiles), and the whiskers extend from the upper and lower hinge to the largest and smallest values no further than 1.5 times the interquartile range from the hinge. Data beyond the end of the whiskers are outliers and are plotted individually.

We then investigated the relative proportions of grains making up the different groups for each of the samples (Figure 7a). Several patterns can be observed. Firstly, there are between-sample differences for the samples from the same site. For example, group 8 was identified in sample HF6008 but absent from samples HF6023 and HF6031. The proportion of grains in groups 1 and 2 vary significantly between samples from HF. Secondly, different sites appear to have different relative proportions in groups. For example, samples from HF generally contain more group 1 and 2 grains compared to the other two sites. In contrast, samples from THD generally have more group 7 grains. Third, all the samples have <10% of grains in groups 6 and 8, and more than half of the grains fall into groups 2 to 5.

## 5. Comparison of OSL Characteristics between Different DRC Groups

To investigate whether different DRC groups had distinctive OSL behaviors (other than their differences in saturation dose) that could be used to distinguish them from each other, we compared the OSL sensitivities (T_n_) and decay curve shapes (FR) of the grains from different groups. The T_n_ of individual grains of different groups for the samples from different sites were compared and are shown in boxplots in Figure 7b. It shows that all groups contain grains with a wide range of sensitivities. There is no distinctive difference in the range of the sensitivities for different groups from the same site. However, it appears that the HF and VISO samples tend to have a larger number of brighter grains in the high-number groups associated with higher saturation doses than THD.

We also investigated the OSL decay shape of grains from different groups. Figure 7c shows the distribution of fast ratios for grains for different groups of different sites in boxplots. No distinctive difference in the range of fast ratios was observed between different groups and sites. However, the fast ratios for the grains from site THD are generally higher than those from the other two sites. The results of Figure 7b and c suggest that there is no discernible correlation between the sensitivity and decay curve shapes of the OSL signals and the shape of DRCs.

**Figure 8:**
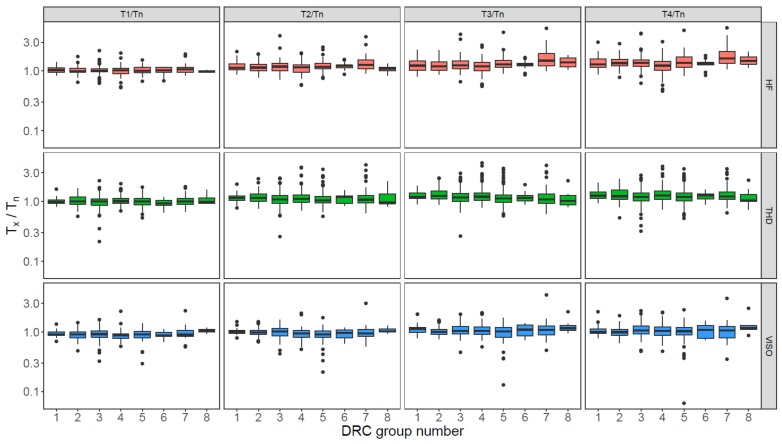
Boxplots showing the ratios between test-dose signals (T_x_) of the second to fifth SAR cycles (T_1_–T_4_) and the first cycle (T_n_) for grains from different groups and different sites (shown in different rows and colors). The center lines in each of the boxes show the data median. Boxes show the first and third quartiles (the 25th and 75th percentiles), and the whiskers extend from the upper and lower hinge to the largest and smallest values no further than 1.5 times the interquartile range from the hinge. Data beyond the end of the whiskers are outliers and are plotted individually.

To check if different groups of grains had different extents of sensitivity change during SAR measurements, we compared the T_x_/T_n_ values for the first five SAR cycles (similar to those shown in Figure 5) for the grains from different groups for each site (Figure 8). Again, no discernible difference in the extent of sensitivity changes among different DRC groups was observed.

## 6. Discussions and Conclusions

Based on single-grain measurements of seven samples from three sites on different continents, we demonstrated that the OSL signal sensitivity and decay shape are highly variable from grain to grain and site to site (Figure 1). Comparisons between the OSL signal sensitivity and decay curve shape suggest that the OSL signals of brighter grains may have faster decay rates (Figure 2), probably indicating that these grains are more dominated by the fast component. However, the brighter and fast component-dominant grains appear to have no advantage over dimmer grains in terms of SAR performance (i.e., recuperation and reproducibility) (Figure 3), and our results suggest that both bright and dim grains could behave poorly during SAR measurements. One of the explanations for this is that the observed variability in the OSL shape does not entirely reflect the physical behavior of the grains. It has been suggested that variation in the OSL decay rate could be caused by the differences in the effective stimulation power when the laser hits the surfaces of different grains [19]. However, previous studies have reported that the grains with fast-decaying OSL signals yield improved results in D_e_ estimation [10,11,44], indicating that the decay shape of laser-based OSL curves does reflect the physical behavior of the grains to some extent. Furthermore, if the stimulation mode is the primary cause, then we should see a similar correlation between the FR and T_n_ for different samples, which is not the case, as shown in Figure 2.

Large variation is observed in the DRCs for different grains, and the extent of variation appears to be similar for different sites and independent of measurement conditions (e.g., preheating temperature) (Figure 4). Although the grains from different sites show different extents of sensitivity change through SAR measurement cycles (Figure 5), the test dose used during the SAR procedure appears to be able to successfully monitor and correct for sensitivity changes for many grains, even though the sensitivity of some may change by >100% from cycle to cycle. Furthermore, there appears to be no correlation between the extent of the sensitivity change and the inherent brightness of the OSL signals or their decay curve shape (Figure 5 and Figure S1).

We analyzed 4500 quartz grains extracted from these sites, and about eight groups were identified and each group shared a similar DRC (Figure 6). Based on the establishment of an SGC for each group, we found that many of the grains (~50%) fall into groups 1 to 3, which are associated with low saturation doses (D_0_ < 50 Gy). Groups 4 to 6, making up 40% to 50% of the accepted grains, have higher saturation doses (D_0_ from ~70 to 110 Gy), and <5% of the grains fall into groups 7 and 8 that have D_0_ values of up to ~200 Gy. Among the analyzed grains, only one ‘super-grain’ with a D_0_ value as high as ~600 Gy was identified. Our results suggest that more than 99% of the grains have D_0_ value <200 Gy, restricting the dating samples with natural doses higher than ~400 Gy (2D_0_) when using the conventional SAR method [45], in which D_e_ is determined for individual grains based on their corresponding DRCs.

Furthermore, since nearly half of the quartz grains have very low D_0_ values (~30–50 Gy), it may result in “truncated” D_e_ distributions (and, hence, D_e_ underestimation) for samples with natural doses >100 Gy [21]. As a result, caution should be taken when dating samples with a large proportion of early saturation grains (e.g., [9,10,11,46]). For this case, it would be important to identify only the grains that have relatively larger D_0_ values to estimate D_e_ values. To overcome this problem, previous studies suggested the selection of grains based on a range of different D_0_ thresholds (e.g., [10,11,12,47]). However, this method has limitations if the D_0_ values are obtained by fitting a few regenerative dose points for each grain, because they strongly depend on measurement uncertainties (e.g., especially for the signal from the largest regenerative dose) and strategy (e.g., the number and range of regenerative doses applied) [21]. This method is also not straightforward when a large number of grains are analyzed and different grains follow different growth patterns. For example, some grains may require a single-saturating exponential plus a linear component to fit their data (in this case, the D_0_ value does not reflect the saturation dose), and some grains may require a double-saturating exponential function (with two different D_0_ values, and only the larger one reflects the saturation dose), which makes the comparison of D_0_ values not straightforward. In contrast, we demonstrated that it is possible to establish SGCs for different groups of quartz [48], which means that one can use the scaling factors of individual grains obtained from SGC analysis to normalize their corresponding natural signals and then apply the new method of Li et al. [21], which involves analyzing the re-normalized L_n_/T_n_ values for grains sharing the same SGC (i.e., from the same group) and projecting the overall estimate (e.g., weighted mean) of L_n_/T_n_ values onto the corresponding SGC to estimate the final D_e_. Unlike the conventional method, in which individual L_n_/T_n_ values are projected onto the corresponding DRCs to estimate D_e_ for individual grains, this method does not reject ‘saturated’ grains, so one can avoid the truncation problem and obtain the full distributions of L_n_/T_n_ for individual DRC groups. This allows a reliable D_e_ estimation beyond the conventional limit of ~2D_0_ using the standard SAR procedure. For the cases where the early saturation groups are saturated (i.e., their mean L_n_/T_n_ values are consistent with the saturation levels of corresponding SGCs), only the later saturation groups can yield finite D_e_ values. This means that all the grains from the early saturation groups are rejected from the final D_e_ estimation. This method has been successfully applied to date several archaeological sites from southwest China [48] and Russia [49,50].

## Figures and Tables

**Table 1:** Number of single grains measured, rejected, and accepted for each sample, together with the reasons for their rejection. BG: background, RSE: relative standard error, FOM: figure of merit, RCS: reduced chi-squares.

Description		HF6008	HF6023	HF6031	THD-OSL2	THD-OSL4	THD-OSL6	VISO-OSL1
**Total Measured**		300	200	500	300	3000	300	2500
1. Weak signal	T_n_ < 3xBG	32 (11%)	17 (9%)	68 (14%)	59 (20%)	1031 (34%)	81 (27%)	870 (35%)
	RSE of T_n_ > 20%	36 (12%)	27 (14%)	55 (11%)	59 (20%)	715 (24%)	61 (20%)	673 (27%)
2. Recuperation	>5%	8 (3%)	7 (4%)	26 (5%)	2 (1%)	15 (1%)	2 (1%)	56 (2%)
3. Poor DRC	FOM > 10%	89 (30%)	26 (13%)	117 (23%)	42 (14%)	374 (12%)	29 (10%)	426 (17%)
	RCS > 5	51 (17%)	45 (23%)	114 (23%)	12 (4%)	520 (17%)	2 (1%)	115 (5%)
Total rejected		216 (%)	122 (61%)	380 (76%)	174 (58%)	2655 (89%)	175 (58%)	2140 (86%)
Total accepted		84 (%)	78 (39%)	120 (24%)	126 (42%)	345 (12%)	125 (42%)	268 (11%)

## Supplementary Materials

The following are available online at https://www.mdpi.com/2409-9279/3/1/2/s1. Figure S1, Ratios between test-dose signals (T_x_) of the 2nd–5th SAR cycles (T_1_–T_4_) and the first cycle (T_n_) plotted against fast ratio for grains from different sites (shown in different rows); Figure S2, Radial plots showing the ratios between the LS-normalised L_x_/T_x_ values and the expected values based on the best-fit SGC shown in Figure 6a; Table S1, The single-aliquot regenerative-dose (SAR) procedure for single grain.
