# Supplementary material for: Single-Grain Quartz OSL Characteristics: Testing for Correlations within and between Sites in Asia, Europe and Africa
Source: Methods Protoc. 2019 Dec 26;3(1):2. doi: 10.3390/mps3010002 (PMC7189676; doi:10.3390/mps3010002)
Supplement: Supplementary file 1 [file mps-03-00002-s001.pdf]

Table S1. The single-aliquot regenerative-dose (SAR) procedure for single grain.

Step	Treatment	Observed
1	Give dose, D_i	
2	Preheat (240°C for 10 s)	
3	Single-grain stimulation with green laser for 2 s at 125°C	L_x
4	Give test dose, D_t (10 Gy)	
5	Cut-heat to 180°C	
6	Single-grain stimulation with green laser for 2 s at 125°C	T_x
7	Blue LED bleach for 40 s at 280°C	
8	Return to step 1	

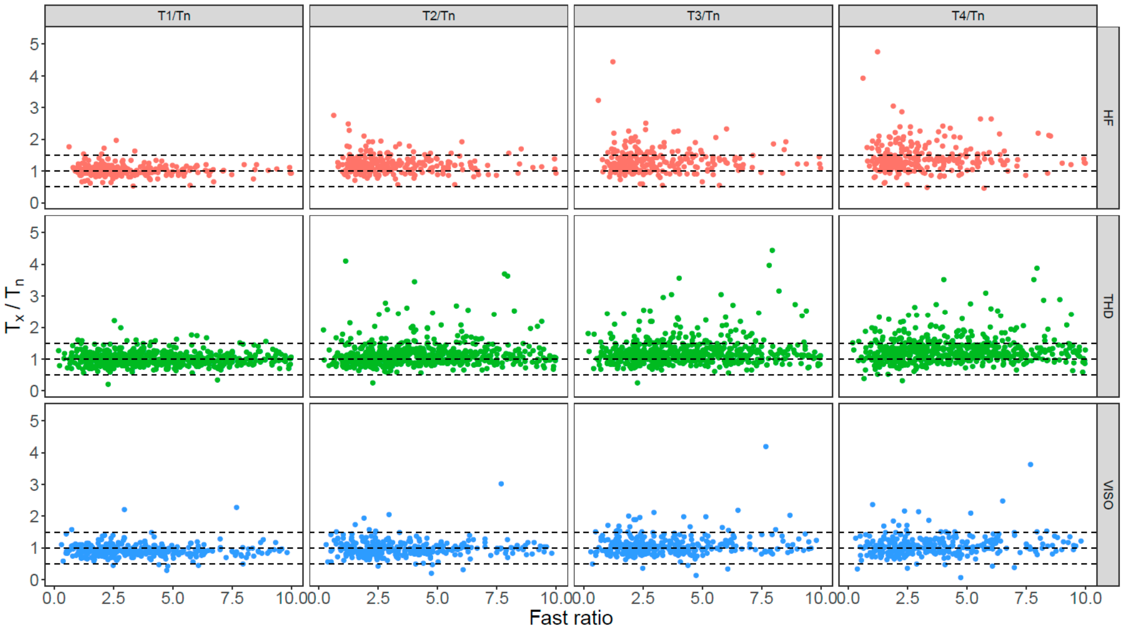

Figure S1. Ratios between test-dose signals (T_x) of the 2nd–5th SAR cycles (T_1–T_4) and the first cycle (T_n) plotted against fast ratio for grains from different sites (shown in different rows). The dashed horizontal lines represents values at 0.5, 1.0 and 1.5, respectively.

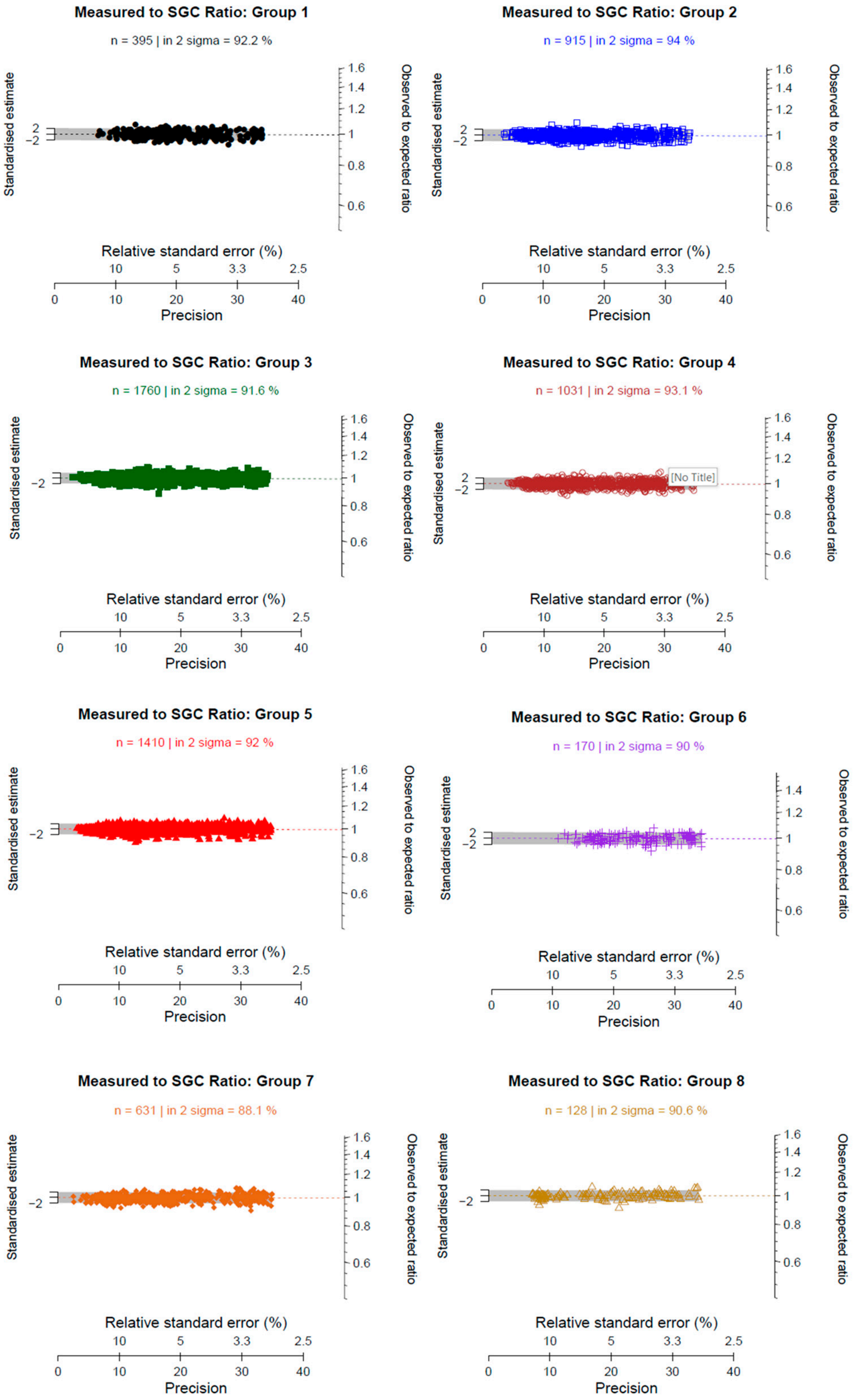

Figure S2. Radial plots showing the ratios between the LS-normalised L_x/T_x values and the expected values based on the best-fit SGC shown in Fig. 6a; the shaded band captures the 2σ range from unity. The total number of grains (n) and percentage falling inside the 2σ band are shown at the top of the plot for each Group.
